# Evaluation of *E. coli Nissle1917* derived metabolites in modulating key mediator genes of the TLR signaling pathway

**DOI:** 10.1186/s13104-021-05568-x

**Published:** 2021-04-26

**Authors:** Sheyda Damoogh, Mehrad Vosough, Shima Hadifar, Masoumeh Rasoli, Ali Gorjipour, Sarvenaz Falsafi, Ava Behrouzi

**Affiliations:** 1 Department of Microbiology, Faculty of Advanced Science and Technology, Tehran Medical Science, Islamic Azad University, Tehran, Iran; 2 Department of Biology, Faculty of Science, Nourdanesh Institute of Higher Education, Isfahan, Iran; 3 Department of Mycobacteriology and Pulmonary Research, Pasteur Institute of Iran, Tehran, Iran; 4 Department of Basic and Applied Science for Engineering, Civil and Industrial Engineering Faculty, Sapienza University of Rome, Rome, Italy

**Keywords:** *EcN*, Inflammatory bowel diseases, Probiotics, TLR signaling

## Abstract

**Objective:**

Gut-microbiota plays key roles in many aspects like the health and illness of humans. It's well proved that modification of gut microbiota by probiotics is useful for improving inflammatory bowel disease (IBD) conditions. According to recent studies, different types of bacterial metabolites can affect immune cells and inflammation conditions. The present study aimed to evaluate the anti-inflammatory effects of metabolites of *E. coli* Nissle1917.

**Results:**

The cell-free supernatant could modulate TNF-α production and affected many crucial mediators in the Toll-like receptor (TLR) signaling pathway. Also, supernatant showed significant dose-dependent properties in this regard. In this study, the TLR signaling pathway was found among probable mechanisms by which probiotics can affect inflammatory situations. These findings provide additional evidence on the use of probiotic metabolites for inhibiting and down-regulating numerous key mediator factors in the TLR signaling pathway. Aberrant or dysfunctional TLR signaling contributes to the development of acute and chronic intestinal inflammatory pathways in IBD. Therefore, finding a component that can affect this process might be considered for therapeutic targets in IBD patients.

**Supplementary Information:**

The online version contains supplementary material available at 10.1186/s13104-021-05568-x.

## Introduction

Inflammatory bowel disease (IBD) is an umbrella term for a group of intestinal disorders that cause prolonged inflammation of the digestive tract. In general, the gut bacterial population has a crucial effect on either the development or recurrence of the IBD [1]. Today, IBD treatment is based on using anti-inflammatory drugs, immune modulators substances, and surgery, which may bring a heavy burden on the therapeutic systems [2]. Some researchers reported that animal models without intestinal bacteria flora did not reveal the expansion of inflammatory diseases such as IBD [3]. However, several studies showed that modifying gut microbiota with probiotics can help improve the IBD condition. Also, prescribing a high concentration of probiotics can end in extensive severity of the inflammatory condition in the intestine of IBD patients [4, 5]. Toll-like receptors (TLRs), as a member of the pattern recognition receptors (PRRs) families, play an indispensable role in inducing anti-inflammatory or inflammatory response and subsequent activation of adaptive immune responses [6]. TLRs are divided into cell membrane TLRs, which are expressed on the surface of cells (including heterodimers of TLR2 with TLR1 and TLR6 and TLR4-5–6) [7] and intracellular TLRs, which are localized to the endosome, lysosomes, or to endoplasmic reticulum (ER) [8]. TLR signaling pathways include MyD88-dependent pathway that used all TLRs (except for TLR3) and can lead to the release of many different inflammatory cytokines [9]. The TRIF-dependent pathway used through TLR3 and 4 can lead to induction of interferon type-1 [10]. TLR signaling pathway has a key role in the innate immune system such that alteration of TLR expression can contribute to IBD. Therefore, aberrant or dysfunctional TLR signaling may contribute to the development of acute and chronic intestinal inflammatory pathways in IBD and impair intestinal homeostasis [11].

*Escherichia coli* Nissle 1917 (EcN; O6: K5: H1), as a probiotic, is attributed to a non-pathogenic and commensal *E. coli*. EcN is used to treat several inflammatory diseases like ulcerative colitis (UC) and Crohn’s disease (CD). The wide use of this strain is because probiotic treatment can promote an anti-inflammatory response. EcN has a positive effect on intestinal homeostasis and microbial balance. In addition, several studies confirmed the therapeutic benefits of this strain [12].

The presence of the metabolites and boosting their population are recommended as effective controlling strategies in IBD studies. Accordingly, in this study, we evaluated the effect of *E. coli Nissle1917* metabolites on TLR signaling gene expression in inflammatory model cell culture. Overall, proposing some microorganisms that are useful for either treatment or identification of different diseases plays a key role in developing next-generation medicine.

## Main text

### Materials and methods

#### *E. coli Nissle1917* culture and preparation of cell-free supernatant

*Escherichia coli* Nissle 191 strain was grown in Lauria-Bertani (LB) broth overnight at 37 ℃ with shaking (150 rpm). The cell-free supernatant was prepared according to the method proposed by Ogunbanwo [13]. *E. coli* Nissle1917 was grown in LB broth for 24 h at 37 ℃. Then, cell-free supernatant was prepared by centrifuging the culture at 20,000 rcf at 4 ℃ for 20 min. Finally, for removing bacterial bodies, they were filtered through 0.2 μm filters [14].

#### Cell culture and create an IBD model cell

The HT-29 was cultured in high glucose Dulbecco’s modified eagle’s medium (HDMEM), which was supplemented with 10% fetal bovine serum (FBS) and 1% penicillin–streptomycin and non-essential amino acids in six-well plates at 37 ℃ in 5% CO2. To mimic inflammatory conditions like IBD circumstance, the culture was treated with pro-inflammatory cytokines. The culture was maintained for 4 weeks to assess monolayer conditions (much more similar to intestinal conditions). In week 3, it was treated with pro-inflammatory cytokines: 10 ng/ml recombinant human IL-1β or 10 ng/ml recombinant human TNFα [15]. After 4 weeks, when HT-29 monolayer was created and the existence of inflammation condition (same as the IBD conditions) was confirmed, the cell line was treated with the minimum inhibitory concentration of *E. coli* Nissle 1917 supernatant and an equal volume of PBS was used as a control well.

#### MTT assay

To evaluate the effect of *E. coli* Nissle1917 metabolites on the viability of HT-29 cells, an MTT assay was performed. HT-29 was treated with a minimum inhibitory concentration of *E. coli* Nissle1917 metabolites and incubated for 24, 48, and 72 h. Next, the MTT solution (5 mg/ml) was added to each well and incubated at 37 for 3 h. After 3 h, the supernatants were removed and blue crystals were solubilized with dimethylsulfoxide (DMSO). Finally, absorbance was measured at a wavelength of 570 nm [16, 17].

#### RNA extraction, cDNA synthesis, and real-time PCR

Total RNA was extracted from treated cells and control cells. After 48 h and confirming their quality by TRIzol reagent, cDNA was synthesized by SCRIPT cDNA Synthesis Kit (Jena Bioscience) according to the manufacturer’s instructions (https://www.jenabioscience.com/). Real-time PCR was performed based on the SYBRGreen method. A sequence of primers is shown in Table 1.

**Table 1:** Sequence of primers used in qPCR in cell line

Genes Name	Forward Sequence (5′-3′)	Reverse Sequence (5′-3′)	Refs.
TLR-1	GCCCAAGGAAAAGAGCAAAC	AAGCAGCAATATCAACAGGAG	[43]
TLR-2	TCTCCCATTTCCGTCTTTTT	GGTCTTGGTGTTCATTATCTTC	[43]
TLR-3	TAAACTGAACCATGCACTCT	TATGACGAAAGGCACCTATC	[18]
TLR-4	GAAGCTGGTGGCTGTGGA	GATGTAGAACCCGCAAG	[18]
TLR-5	TTGCTCAAACACCTGGACAC	CTGCTCACAAGACAAACGAT	[18]
TLR-6	GTGCCATTACGAACTCTA	TTGTTGGGAATGCTGTT	[18]
TLR-7	CTGACCACTGTCCCTGAG	AACCCACCAGACAAACCA	[18]
TLR-8	AACATCAGCAAGACCCAT	GACTCCTTCATTCTCCCT	[18]
TLR-9	CGCCAACGCCCTCAAGACA	GGCGCTTACATCTAGTATTTGC	[18]
Myd88	TGGCCTTGTTAGACCGTGA	AAGTATTTCTGGCAGTCCTCCTC	[44]
IRAK1	AGGTTTCGTCACCCAAACATT	CGGGCTGTACCCAGAAGGA	[45]
IRAK4	CCTGACTCCTCAAGTCCAGAA	ACAGAAATGGGTCGTTCATCAAA	[46]
MAPK	GACGAGGAGCTTATGGTTCTGT	TTTTCATCCACTGTTGACCGAA	[47]
NFkB	GTCAAAAACGCCACCTCTCAA	CTCGCATGGAATTTGGAACCG	[22]
TAB2	CTCCTGGTGGTACAACTCGAC	TGATTTGGCTGTTGAGATGAGG	[48]
IFN-β	CAACTTGCTTGGATTCCTACAAAG	TATTCAAGCCTCCCATTCAATTG	[48]
Β-actin	AACCGTGAAAAGATGACCCAGAT	CACAGCCTGGATGGCTACGT	[19]
GAPDH	AACGGGAAGCTTGTCATCAATGGAAA	GCATCAGCAGAGGGGGCAGAG	[18]

#### Analysis of data

The ΔΔct method was carried out for evaluating and analysis of relative gene expression. We also used different housekeeping genes (Β-actin, GAPDH) for choosing a better reference gene. GraphPad Prism 8.0 was used for calculating changes in gene expression and analysis of cytokine as well. A *P-*value of less than 0.05 was considered statistically significant.

### Results

#### MTT assay

**Fig. 1:**
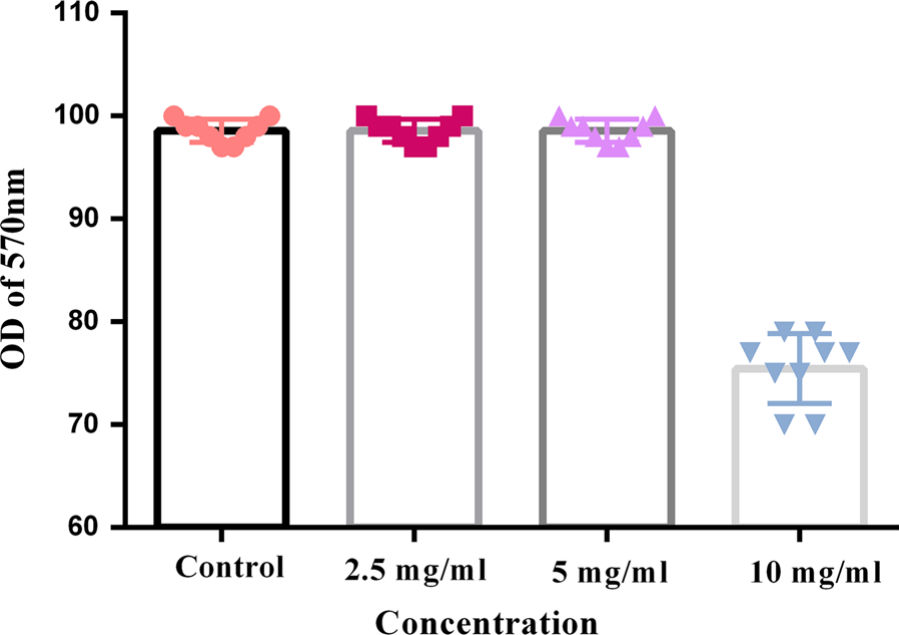
MTT assay: Evaluation of viable cells in present of different concentration of cell-free supernatant

The efficiency of different concentrations of bacterial cell-free supernatants on the viability of cells was evaluated after 48 h by the MTT assay. Three concentrations of 2.5, 5, and 10 mg/ml were used for this purpose. The concentration level of 10 mg/ml indicated a significant inhibitory effect on HT-29 cell line viability compared to the control sample (p < 0.05). Meanwhile, other concentrations (i.e. 2.5 and 5 mg/ml) did not show a significant inhibitory effect, which indicates that the supernatant of the *E. coli Nissle 1917*, as a probiotic strain, can affect the growth rate of cancerous cell lines. In addition, the efficiency of the cell-free supernatant was dose-dependent, which allows considering it in future studies on cancer aspects, especially gastrointestinal cancer. Studies that used some probiotic bacteria confirmed that they could reduce inflammation in cancer patients [18]. However, the 5 mg/ml concentration was chosen for continuing the project, because it was intended to evaluate the cell-free supernatant efficiency in total viable cells. Besides, a larger dataset supports this idea that we can modify gene expression without changing its phenotype (Fig. 1).

#### Effects of *E. coli Nissle1917 *metabolites on TLRs gene expression levels

**Fig. 2:**
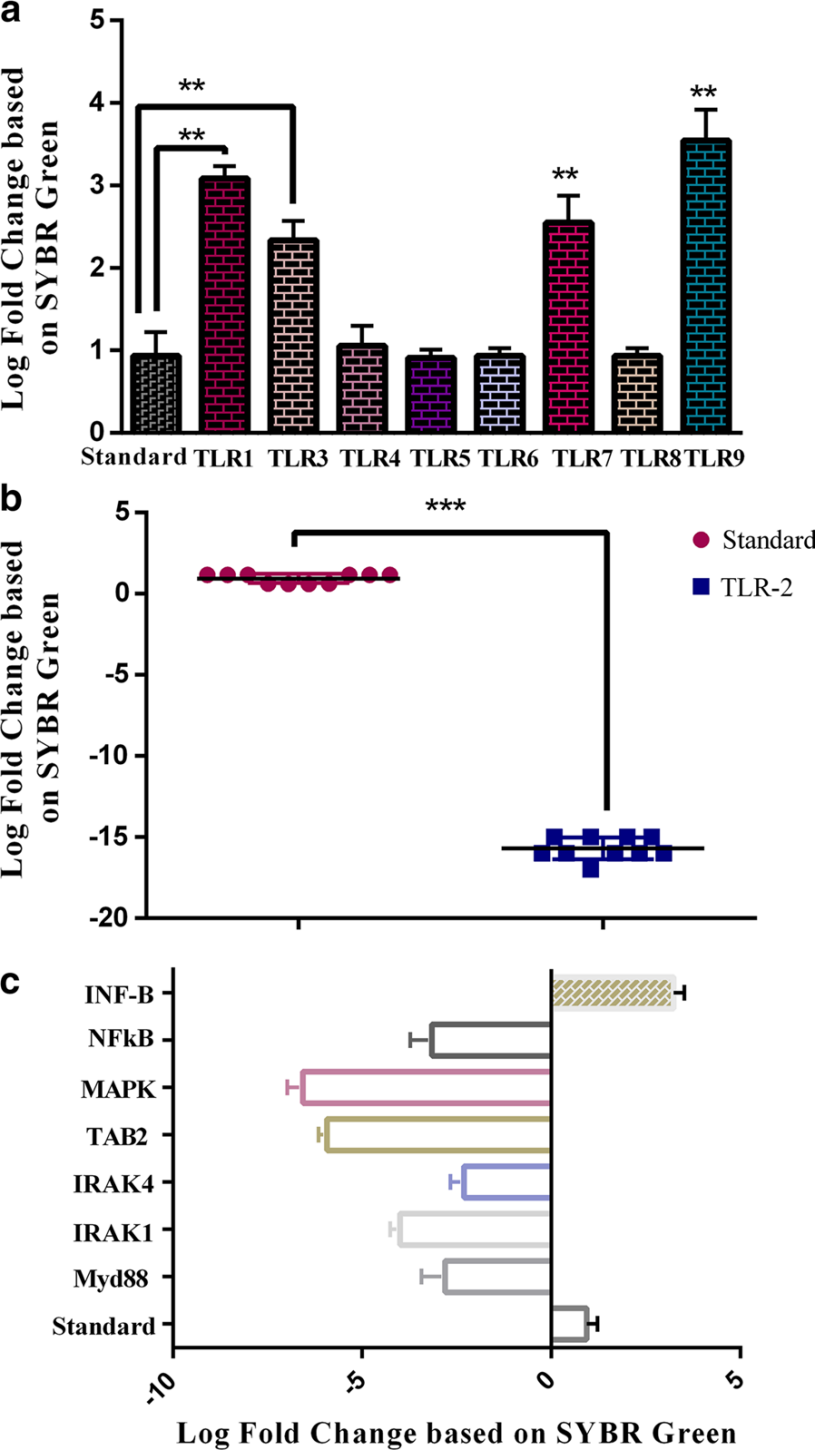
**a** The assessment of cell-free supernatant effect on the TLRs genes in the IBD model by Real Time PCR based on SYBR Green method. *P* < 0.01 were considered statistically significant; **b** TLRs recruit different adaptors like as MyD88, TRIF, TIRAP/MAL, or TRAM. All TLRs can utilize the Myd-88 adaptor and activates NF-kB and MAPKs for induction of inflammatory cytokines. Key genes in this pathway which were evaluated in the study showed by arrows; **c** The evaluation of key mediator expression genes in TLR signaling pathway in present of cell-free supernatant.* P* < 0.01 were considered statistically significant

There is a direct correlation between IBD and microbiota. In this regard, we evaluated the expression levels of TLRs genes involved in IBD [11]. The expression levels of TLR-2 showed a statistically significant decrease (p < 0.05) compared to the control sample, while the expression levels of TLR-1, TLR-3, and TLR-7 were increased. The other TLR genes (i.e. TLR-4, TLR-5, TLR-6, and TLR-8) did not show any change in their expression levels over the control sample. According to the findings, most changes were related to a 15-times reduction in TLR-2 expression. Other changes in expression levels ranged from 2 to 5 times compared to the control sample (Fig. 2a).

#### Effects of *E. coli Nissle1917 *metabolites on Myd88, IRAK1, IRAK4, TAB2, MAP3K, and NFkB gene expression levels

TLRs receptors are located in several cell types such as epithelial cells, macrophages, dendritic cells, T cells, B cells, and endothelial cells [19]. Previous studies that used animal models with a deficiency in MyD88 have confirmed the important role of TLR/MyD88 signaling in the inhibition of intestinal inflammation [20]. Interestingly, all key mediator genes in the TLR signaling pathway that were evaluated in our study, including Myd88, IRAK1, IRAK4, TAB2, MAP3K, and NFkB, showed a significant reduction in the expression level compared to the control sample. However, in contrast, the expression levels of INF-β1 were increased significantly (Fig. 2b and c and Additional file 1: Figure S1).

### Discussion

These days, probiotics are widely using in various pathways for addressing many medical problems [21]. For instance, the emergence of several infectious agents, which are multi-drug-resistant (MDR), has led to public health concerns. Various agents are responsible in this regard, for instance using indiscriminate and inadequate antibiotics [22] or overexpressed efflux pumps, especially in gram-negative bacteria [23]. Therefore, the utilization of probiotics, instead of antibiotics, for treating several diseases has been evaluated [24]. Additionally, other properties of probiotics include prevention and treatment of different cancers [25]. However, one of the innovative functions of probiotics is reducing or treatment of many inflammatory diseases [26]. *E. coli Nissle*1917 has a wide array of intestinal benefits for its hosts. Several antimicrobial agents, such as defensing, can inhibit adhesion and invasion of pathogenic bacteria. EcN, as a probiotic bacteria, can induce immune responses in IBD patients by defensin production [27]. Also, it has a positive effect on the expression (up-regulation) of zonula occludens (ZO) proteins (namely, ZO-1 and ZO-2) and thus repairing leaky gut phenomena [28]. Other favorable effects of EcN are related to reducing pro-inflammatory cytokines such as IL-2, TNFα, and IFNγ and increasing anti-inflammatory cytokines, which are helpful to maintain immunological homeostasis [29]. These properties might be because EcN has a specific LPS without serious immunotoxic properties and it has a strong influence on intestinal immune conditions [30]. However, some studies reported that using live microorganisms can negatively affect patients and in some cases has led to increased inflammatory conditions. Therefore, using the metabolites of microorganisms as a therapeutic agent seems to be a controversial issue. In the present study, the probiotic strain was chosen based on previous studies, which indicates that the anti-inflammatory effects of EcN can decrease inflammatory cytokine levels [29]. On the other hand, the TLR signaling pathway has a crucial effect on the pathogenesis of IBD. Also, it affects the efficiency of treatment in IBD [11]. Several functions have been recognized for TLRs, such as detection of pathogenic agents, detection of various antigens, and bridging the adaptive and innate immunity, which can regulate numerous pathways such as cytokine production, proliferation, and survival [31]. Initiating signaling pathways through TLRs function can stimulate the production of different cytokines and chemokines [32]. As there is a direct association between IBD and some TLR, understanding these pathways provides the opportunity to find out appropriate strategies for improving the treatment of IBD patients [33]. Additionally, there is a direct correlation between the alternation of TLR receptor genes and the composition of the microbiota and people who suffer from IBD have different TLR genes expression against healthy people [34]. Therefore, because of the importance of EcN in intestinal immune responses and the importance of the TLR signaling pathway in immune responses, we evaluated the vital genes in the TLR signaling pathway dysfunction of the immune system. The increased expressions of TLR-2, 4, 8, and 9 have been reported in CD patients while the expression of TLR5 depended on the severity of the disease [35]. In addition, the findings indicated decreased expression of TLR2, while no change was observed in the expression of TLR 4, 5, and 6. Hence, it can be speculated that EcN could improve the inflammatory condition in CD patients due to the lack of LPS (for prompting TLR-4) and flagellin (for prompting TLR-5).

Another speculation is the loss of structure and structural component after centrifuging. Thus, they could not induce some TLR-gene expressions. A previous study demonstrated that inhibition of the TLR2/6 signaling pathway has an indispensable role in the progression of IBD [36]. In this regard, our results revealed decreased TLR-2 expression along with un-changeable TLR6 expression. Hence, we assumed the inhibition of the TLR1/2 pathway. Furthermore, regarding the vital role of TLR1 signaling in mucosal protection [37], this study demonstrated that metabolites of *E. coli* Nissle1917 can stimulate the expression of TLR-1 significantly. Besides, based on a previous study, lack of TLR1 during gastrointestinal infection end up in the activation of chronic immunity [38], which might confirm the fact that TLR1 and its signaling pathway can inhibit inflammation in IBD patients [11]. TLR4 is another receptor that is important in IBD because it has a preliminary role in the inflammatory response against pathogenic bacteria. Therefore, it might be effective on UC diseases [39]. Nevertheless, in the present study, TLR-4 expression did not show any change. Gibson et al. demonstrated that TLR-2 had a protective effect against different dangerous agents and TLR-2 could maintain mucosal integrity against increased inflammatory responses by the TLR-4 pathway [40]. A previous study indicated the conspicuous down-regulation of TLR3 and TLR7 in epithelial cells of patients who suffer from IBD [41]. Besides, other studies showed a direct correlation between TLR3TLR-7 genetic variation and the severity of UC disease [42, 49].

### Conclusion

Finally, mechanistic exploration of the function of the component in different pathways can help design innovative therapeutic agents in several medical aspects.

### Limitation

It is necessary to mention some limitations and biases of our study, including evaluating a few number of cytokines, which should be addressed in future studies.

## Supplementary Information

**Additional file 1: Figure S1.** Important key mediator genes in Toll-Like Receptors (TLRs) signaling pathway.

## Data Availability

All data generated or analysed during this study are included in this published article and its supplementary information files.

## Abbreviations

PRRs: Pattern recognition receptors
TLR: Toll-like receptors
MYD88: Myeloid differentiation primary response 88
IRAK: Interleukin receptor-associated kinase
IBD: Inflammatory bowel disease
CD: Crohn's disease
UC: Ulcerative colitis
EcN: *E. coli Nissle1917*
IFN: Interferon
TRIF: TIR-domain-containing adapter-inducing interferon-β
NFkB: Nuclear kappa beta
UEV1A: Ubiquitin-conjugating enzyme E2 variant 1
MAP: Mitogen-activated protein
IkB: Inhibitor of nuclear factor-kB
TAK: Transforming-growth factor-k-activated kinase
