# Supplementary figures and images for: Evaluation of E. coli Nissle1917 derived metabolites in modulating key mediator genes of the TLR signaling pathway
Source: BMC Res Notes. 2021 Apr 26;14:156. doi: 10.1186/s13104-021-05568-x (PMC8077910; doi:10.1186/s13104-021-05568-x)

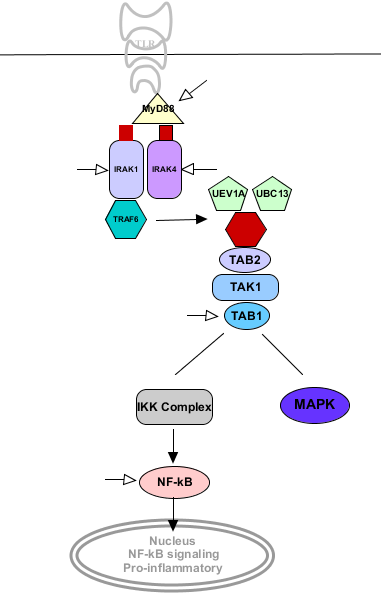

Supplement: Supplementary file 1 — Additional file 1: Figure S1. Important key mediator genes in Toll-Like Receptors (TLRs) signaling pathway. [file 13104_2021_5568_MOESM1_ESM.png]
